# The yield of a comprehensive investigation protocol for the diagnosis of true idiopathic ventricular fibrillation in a real-life clinical setting

**DOI:** 10.1038/s41598-024-64513-7

**Published:** 2024-06-18

**Authors:** Samuel Lietava, Milan Sepsi, Jana Zidkova, Iva Synkova, Milan Kozak, Lubomir Krivan, Jitka Vlasinova, Svatopluk Richter, Jan Rehor, Petr Kala, Marketa Bebarova, Tomas Novotny

**Affiliations:** 1 Department of Internal Medicine and Cardiology, University Hospital Brno and Faculty of Medicine, Masaryk University, Jihlavska 20, 625 00 Brno, Czech Republic; 2 Centre of Molecular Biology and Genetics, University Hospital Brno and Faculty of Medicine, Masaryk University, Jihlavska 20, 625 00 Brno, Czech Republic; 3 Department of Radiology and Nuclear Medicine, University Hospital Brno and Faculty of Medicine, Masaryk University, Jihlavska 20, 625 00 Brno, Czech Republic; 4 Department of Physiology, Faculty of Medicine, Masaryk University, Kamenice 5, 625 00 Brno, Czech Republic

**Keywords:** Cardiovascular genetics, Ventricular fibrillation

## Abstract

Traditionally, aborted cardiac arrest (ACA) due to documented ventricular fibrillation (VF) in the absence of structural heart disease has been termed idiopathic VF. By careful evaluation, a specific etiology can be found in a substantial proportion of patients. The aim of this survey was to assess the yield of an advanced diagnostic work-up to reveal a causative etiology in a real-life clinical setting. Patients from the University Hospital Brno’s ACA database were analyzed (514 patients in total). Forty-six patients (31 males) fulfilled the inclusion criteria, which were: (1) absence of structural pathology on echocardiography; (2) absence of coronary artery disease; and (3) absence of reversible cause of ACA. The diagnostic work-up consisted in cardiac magnetic resonance imaging, stress testing, sodium channel blocker challenge, and genetic testing according to the availability of the method and patient compliance. A specific disease was found in 17 individuals (37.0%), although at least one diagnostic step was refused by 13 patients (28.3%). True idiopathic VF was confirmed in 7 patients (15.2%), for whom the entire diagnostic work-up did not reveal any specific pathology. Our real-life survey shows that, even with an incomplete diagnostic work-up (due to the unavailability of a particular method or variable patient compliance), a specific diagnosis can be identified in more than one third of the cases of “idiopathic” VF, which can thus enable targeted treatment and family screening.

## Introduction

Sudden cardiac arrest (SCA), which is defined as a fatal unexpected event due to a cardiovascular cause, is a major public health problem^1^. In Europe, SCA accounts for 10–20% of all deaths^2–4^. Coronary artery disease (CAD) is the most common etiology of SCA^5,6^, followed by cardiomyopathies, anatomical heart abnormalities, and hereditary arrhythmic syndromes^2,7,8^. Nevertheless, to reveal the latter’s etiology a comprehensive cardiac assessment is necessary as electrocardiographic (ECG) abnormalities may be subtle or intermittent.

Traditionally, aborted cardiac arrest (ACA) due to documented ventricular fibrillation (VF) in the absence of structural heart disease has been termed idiopathic VF^9^. Using a thorough investigative algorithm, a definitive diagnosis can be made in substantial proportion of VF-based ACA survivors. This work-up includes (in addition to the standard clinical and paraclinical examinations) a repeated 12-leads electrocardiogram, cardiac imaging (echocardiography and cardiac magnetic resonance (CMR)), coronary angiography, exercise stress test and pharmacological challenge^10^. Thus, currently the diagnosis of idiopathic VF is reserved for cases in which the examinations mentioned above do not reveal positive results. However, not every real-life ACA survivor has been investigated in a comprehensive manner.

The aim of this survey was to assess the yield of advanced cardiological investigations to reveal a causative etiology in a real-life clinical setting where some investigation modalities are not always available and patient compliance is variable.

## Methods

### Study population

Patients were enrolled in this study if they were survivors of ACA with documented VF and they also fulfilled the following criteria: (1) absence of structural pathology on echocardiography; (2) absence of CAD (coronary arteries were without stenosis or with stenosis of < 50%); (3) absence of reversible cause as marked hypokalemia, drug overdose or commotion cordis. Patients were allowed to have transient left ventricular dysfunction or QT interval prolongation immediately after cardiac arrest if these findings normalized promptly.

In this single-center real-life survey, all consecutive patients fulfilling the criteria defined above who were referred between January 2007 and December 2022 to the Department of Internal Medicine and Cardiology, University Hospital Brno, were analyzed retrospectively. The study conformed to the principles outlined in the Declaration of Helsinki. For the clinical part of this survey, permission from ethics committees was not needed, as all examinations were performed by clinical standards in accordance with the guidelines of the European and Czech cardiology societies. Nevertheless, all patients consented separately to each individual investigation.

The genetic part of the study was approved by the Multicenter Ethical Committee, University Hospital Brno (Brno, Czech Republic). All participants signed a written informed consent form.

### Data collection

During index hospitalization, information on medical history, a physical examination, blood tests, baseline 12-lead ECG, continuous ECG monitoring during hospitalization, 2D transthoracic echocardiography (2D-TTE) and coronary angiography were obtained in all the cases. CMR was intended for all patients, nevertheless it was performed according to its availability for certain patients before they received an implantable cardioverter defibrillator (ICD) for secondary prevention. The CMR findings were analyzed by an imaging expert to assess possible signs of arrhythmogenic cardiomyopathy (ACM).

Coronary angiography was considered non-diagnostic of CAD if the coronary arteries were either with no stenosis or if stenosis of < 50% was present. Ergonovine challenge was performed at the discretion of the interventional cardiologist.

2D-TTE findings were considered normal if the ejection fraction of the left ventricle was ≥ 50% (calculated using the Teichholz and Simpson formula) and in the presence of, at most, mild valvar disease.

ECGs recorded during index hospitalization were reviewed by two cardiologists. The ECG recordings were analyzed at a paper speed of 25 mm/s and an amplitude of 10 mm/mV. According to current guidelines, a diagnosis of Brugada syndrome (BrS) was established if a coved ST elevation ≥ 2 mm was documented spontaneously in ≥ 1 lead from V1–2 positioned in the 4th, 3rd and 2nd intercostal space^10^. Long QT syndrome (LQTS) was suspected if the QT interval was ≥ 480 ms corrected to heart rate using the Fridericia and Bazett formulas.

Further investigations (exercise stress test and sodium channel blocker challenge using ajmaline) were offered to all patients after recovery, usually not earlier than 4 weeks after the ACA. If they expressed their oral consent, then in most cases at first a bicycle exercise stress test was performed. Standard 12-lead ECGs with Mason–Likar electrode positions were used. The initial exercise stress was set at 0.5 W/kg and increased by 0.5 W/kg every 3 min up to achieving 75–85% of age-specific maximal heart rate, which was defined as 220 beats per minute minus age in years. The LQTS was diagnosed if repeated QT interval prolongation (QTc ≥ 480 ms) was observed or the diagnostic (Schwartz) score was > 3. Catecholamine polymorphic ventricular tachycardia (CPVT) was diagnosed if bidirectional or polymorphic ventricular tachycardia was provoked during the exercise stress test.

Sodium channel blocker challenge was another part of the diagnostic work-up, while the sequence of stress test and pharmacological challenge was at the discretion of individual physicians. Ajmaline was administered intravenously at a dose of 1 mg/kg with a speed of 10 mg/min. BrS diagnosis was confirmed if a typical ECG pattern developed (as described above).

The diagnostic work-up of idiopathic VF was considered complete if 12-lead ECG, coronary angiography, 2D-TTE, exercise stress test and sodium channel blocker challenge were performed or if the particular patient refused a certain step of the protocol. The investigative algorithm was stopped at the particular step which led to the establishment of the final diagnosis (i.e. sodium channel blocker challenge was not performed in patients with LQTS or CPVT diagnosis).

### Genetic analysis

Genetic analysis was offered to all patients with a definitive diagnosis of true idiopathic VF (full work-up protocol completed or only stress test or sodium channel blocker challenge missing). Said analysis was performed with next generation sequencing gene panels for both primary electrical diseases of the heart and cardiomyopathies (KAPA HyperChoice (Roche, USA)/MiSeq or NextSeq 500 (Illumina, USA)). Only findings in genes with definitive and strong gene-disease validity were taken into consideration^11^. All detected genetic variants were classified according to the VarSome variant classifier (https://varsome.com) and the ACMG guidelines^12^ as pathogenic/likely pathogenic (P/LP), variants of unknown significance (VUS), or benign/likely benign.

### Statistical analysis

Statistical tests were performed using the Python programming language in the Spyder development environment. Qualitative data were expressed as frequencies and quantitative data as mean ± standard deviation. A two-tailed t-test was used for quantitative variables. The Shapiro–Wilk test was performed to verify normality and Levene’s test was used to verify the agreement of variances**.** If the assumption of normality was not present, a non-parametric test—the Mann–Whitney U-test—was used. This was done for sex and age. Since multiple statistical tests were performed on the same dataset, the Bonferroni correction was used. For qualitative data, a Z-test of proportions was used with Bonferroni’s correction. P < 0.05 or P < 0.02 after Bonferroni’s correction were considered statistically significant.

## Results

**Figure 1:**
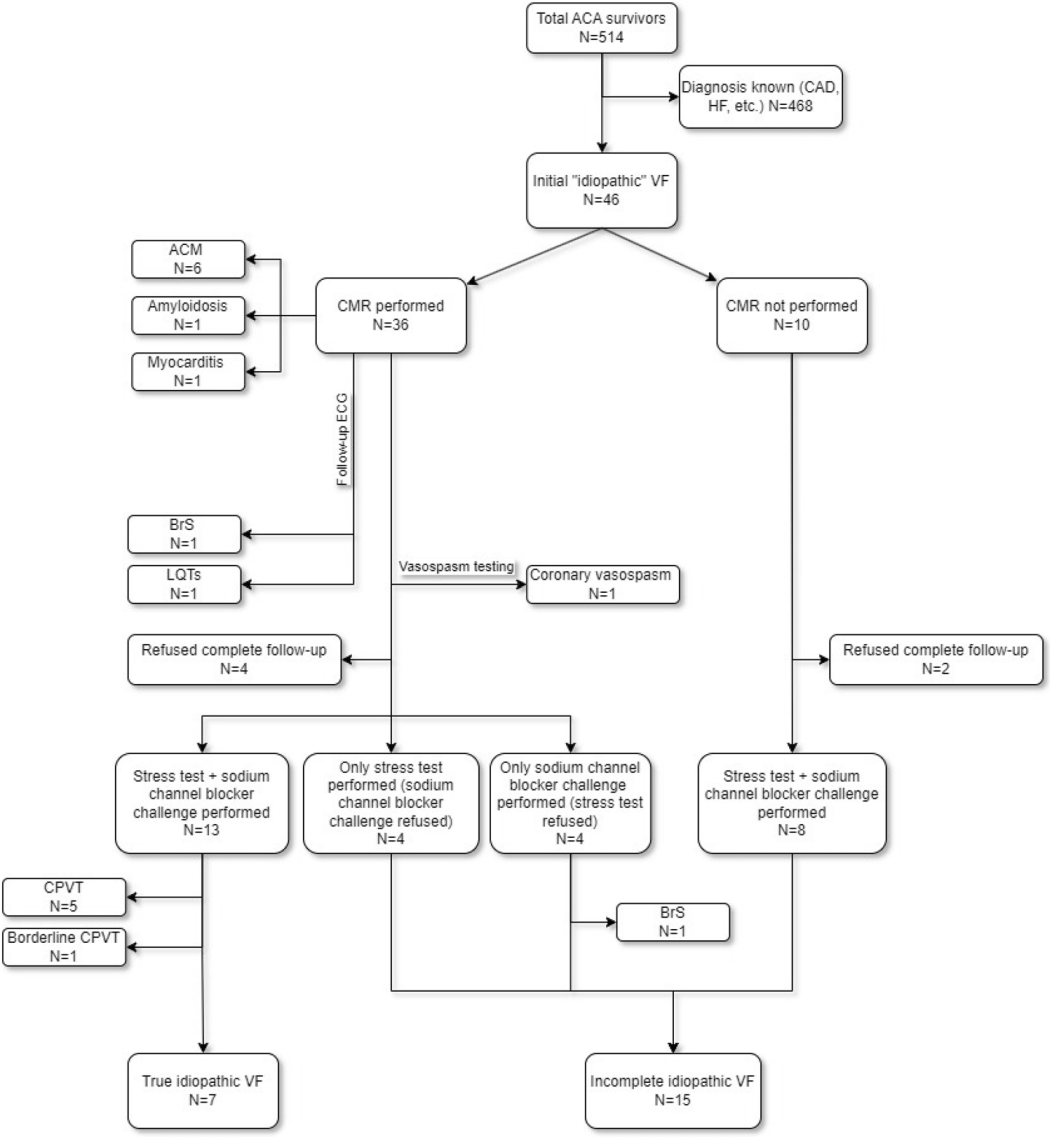
Sequence of the investigations and patient flow in the investigation protocol. *ACA* aborted cardiac arrest, *CAD* coronary artery disease, *HF* heart failure, *VF* ventricular fibrillation, *ACM* arrhythmic cardiomyopathy, *CMR* cardiac magnetic resonance, *ECG* electrocardiogram, *BrS* Brugada syndrome, *LQTS* long QT syndrome, *CPVT* catecholaminergic polymorphic ventricular tachycardia.

During the analyzed period, of the 514 ACA survivors in the registry 46 patients fulfilled the inclusion criteria. Normal findings on resting ECG, 2D-TTE and coronary angiography were observed in all of these subjects. Figure 1 shows a schema of the investigation protocol and the flow of individual cases. In the event of a conclusive diagnosis at any step of the protocol, further investigations were not performed. As the order of examination was not strictly given, the numbers of patients who underwent a stress test and sodium channel blocker challenge are not equal. The characteristics of the population are summarized in Table 1.

**Table 1:** Population characteristics.

	Overall N = 46	≤ 40 years N = 28	> 40 years N = 18	Males N = 31	Females N = 15
Age	36.4 ± 15.3	26.3 ± 9.1	51.5 ± 9.4	36.6 ± 15.7	35.9 ± 15.0
ECG	46 (100%)	28 (100%)	18 (100%)	31 (100%)	15 (100%)
Imaging methods					
2D-TTE	46 (100%)	28 (100%)	18 (100%)	31 (100%)	15 (100%)
LVEF [%]	60.1 ± 7.0	59.6 ± 6.7	61.0 ± 7.5	60.5 ± 7.4	59.3 ± 6.2
Coronary angiography	46 (100%)	28 (100%)	18 (100%)	31 (100%)	15 (100%)
CMR	36 (78.3%)	18 (64.3%)	18 (100%)	22 (71.0%)	14 (93.3%)
Stress test	30 (65.2%)	21 (75.0%)	9 (50.0%)	18 (58.1%)	12 (80.0%)
Sodium channel blocker challenge	26 (56.5%)	18 (64.3%)	8 (44.4%)	19 (61.3%)	7 (46.7%)
Refused complete follow-up	13 (28.3%)	6 (21.4%)	7 (38.9%)	8 (25.8%)	5 (33.3%)

*ECG* electrocardiogram, *2D-TTE* 2-dimensional transthoracic echocardiography, *LVEF* left ventricle ejection fraction, *CMR* cardiac magnetic resonance.

In one female, coronary artery vasospasm was suspected and subsequently confirmed by acetylcholine testing.

CMR during index hospitalization was performed in 36 cases (78.3%). According to the CMR findings, 6 patients (13.0%) fulfilled the criteria of ACM, while 1 female was diagnosed with cardiac amyloidosis. Acute myocarditis was detected by CMR in another female. In the remaining individuals, a CMR was not performed as the method was not available (n = 10, 21.7%). All of these cases fit in the period before 2017 and were labelled as “incomplete” idiopathic VF in our survey.

Hereditary arrhythmic syndromes were diagnosed in 8 patients. Based on repetitive ECGs during follow-up, LQTS was diagnosed in 1 female. Five patients (10.9%) had a positive stress test that revealed CPVT. In 2 males (4.3%), Brugada pattern on ECG was observed during a follow-up investigation: in 1 man spontaneously after a VF event during the follow-up, in the other during the sodium channel blocker challenge. In 1 patient, couplets of bidirectional premature ventricular complexes (PVCs) were induced during the stress test; this case was considered as borderline CPVT.

**Figure 2:**
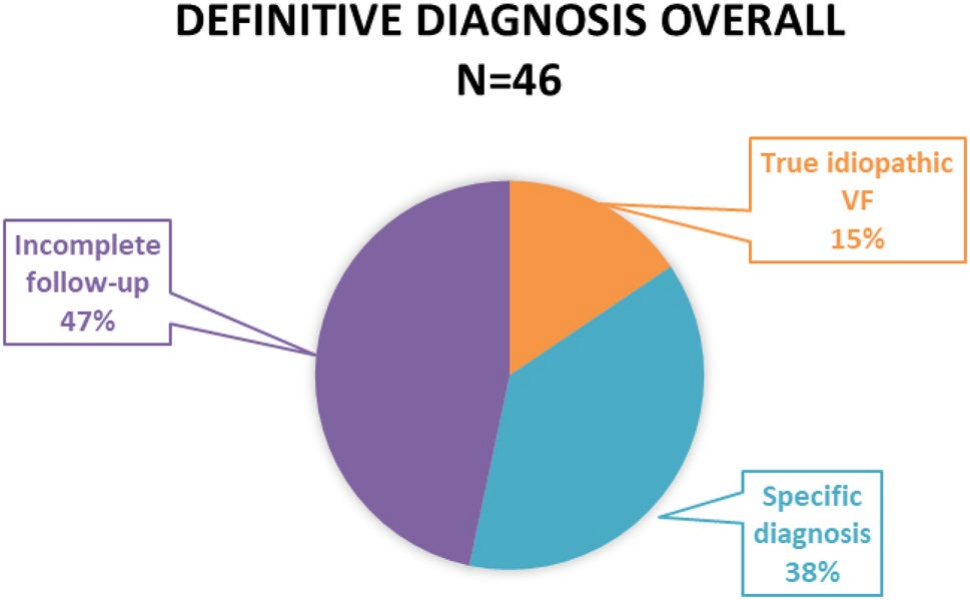
Definitive diagnosis. Incomplete follow-up group contains individuals who either refused all investigations or some step of algorithm is missing. *VF* ventricular fibrillation.

A complete follow-up examination (or any particular step) was refused by 13 patients (28.3%). Both the stress test and pharmacological challenge were refused by 6 patients. Four individuals consented only with the stress test and another 3 only with the pharmacological challenge, and after negative results this group (n = 7, 15.2%) was termed “incomplete” idiopathic VF. A specific diagnosis was found in 17 patients (37.0%). In 7 patients (15.2%), the entire diagnostic algorithm did not reveal any specific pathology and these cases were marked as true idiopathic VF (Fig. 2).

All patients with a diagnosis of true idiopathic VF (n = 7, see Fig. 1, Table 2) gave their consent with genetic analysis. One P/LP variant in the *RYR2* gene (NM_001035.3:c.1244C>T p.(Thr415Ile)) was found in a male patient without any clinical manifestation of CPVT. In 2 other individuals, a variant of uncertain significance (VUS) was found in the following genes: *SCN5A* (NM_000335.5:c.403A>G p.(Met135Val)) in one female, and *MRPL44* (NM_022915.5: c.931A>G p.(Arg311Gly)) in one male.

**Table 2:** Diagnoses based on age and sex.

	Overall N = 46	≤ 40 years N = 28	> 40 years N = 18	P value	Males N = 31	Females N = 15	P value
LQTS	1 (2.2%)	0	1 (5.6%)	NS	0	1 (6.7%)	NS
CPVT	5 (10.9%)	5 (17.9%)	0	NS	3 (9.7%)	2 (13.3%)	NS
Borderline CPVT	1 (2.2%)	0	1 (5.6%)	NS	1 (3.2%)	0	NS
ACM	6 (13.0%)	2 (7.1%)	4 (22.2%)	NS	6 (19.4%)	0	NS
BrS	2 (4.3%)	1 (3.6%)	1 (5.6%)	NS	2 (6.5%)	0	NS
Other	3 (6.5%)	2 (7.1%)	1 (5.6%)	NS	0	3 (20.0%)	0.01
Final diagnosis	17 (37.0%)	10 (35.7%)	7 (38.9%)	NS	11 (35.5%)	6 (40.0%)	NS
Incomplete follow-up	21 (45.6%)	14 (50.0%)	7 (38.9%)	NS	15 (48.4%)	6 (40.0%)	NS
True idiopathic VF	7 (15.2%)	4 (14.3%)	3 (16.7%)	NS	4 (12.9%)	3 (20.0%)	NS

*NS* no significance, *VF* ventricular fibrillation, *LQTS* long QT syndrome, *CPVT* catecholaminergic polymorphic ventricular tachycardia, *ACM* arrhythmic cardiomyopathy, *BrS* Brugada syndrome.

From the group of “incomplete” true idiopathic VF (n = 15, in which one or more examinations from the investigation algorithm is missing, see Fig. 1), 10 patients gave their consent to genetic analysis. In 1 female, a P/LP variant was found in the *FLNC* gene (NM_001458.5:c.3543dup p.(Glu1182Argfs*10)). In another female, a VUS in the *DSP* gene was found (NM_004415.4:c.7412C>T p.(Pro2471Leu)). A VUS in the RYR2 gene (NM_001035.3:c.14201A>G p.(Tyr4734Cys)) was found in one male patient.

In the subgroup of patients missing only CMR in the algorithm (n = 8, see Fig. 1), genetic analysis was performed in all these patients without finding P/LP variants. So genetic analysis by itself did not lead to any particular diagnosis.

**Figure 3:**
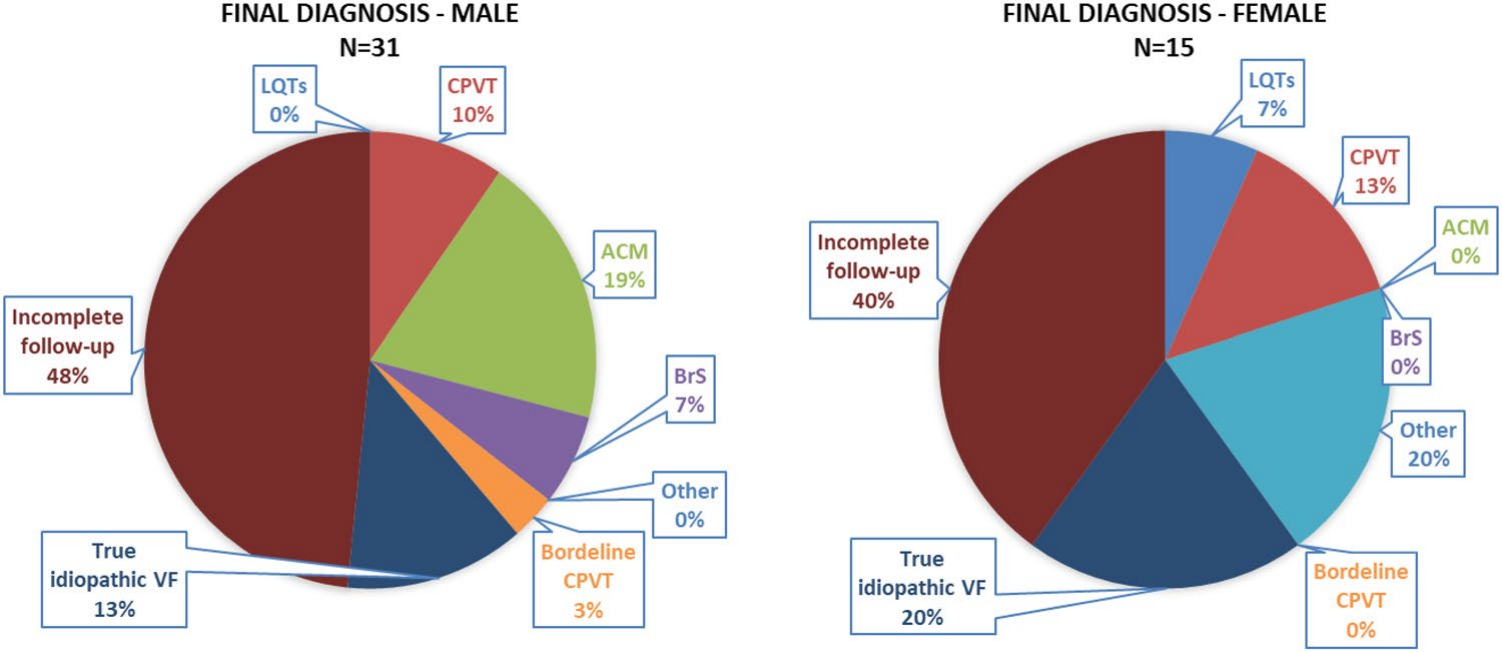
Sex differences in final diagnosis. In females, a statistically significantly higher incidence was observed in the category “Other” (i.e. myocarditis, amyloidosis, coronary vasospasm) as was a trend to a lower incidence of ACM. Incomplete follow-up group contains individuals who either refused all investigations or some step of algorithm is missing. *ACM* arrhythmogenic cardiomyopathy, *LQTS* long QT syndrome, *CPVT* catecholaminergic polymorphic ventricular tachycardia, *ACM* arrhythmic cardiomyopathy, *BrS* Brugada syndrome, *VF* ventricular fibrillation.

**Figure 4:**
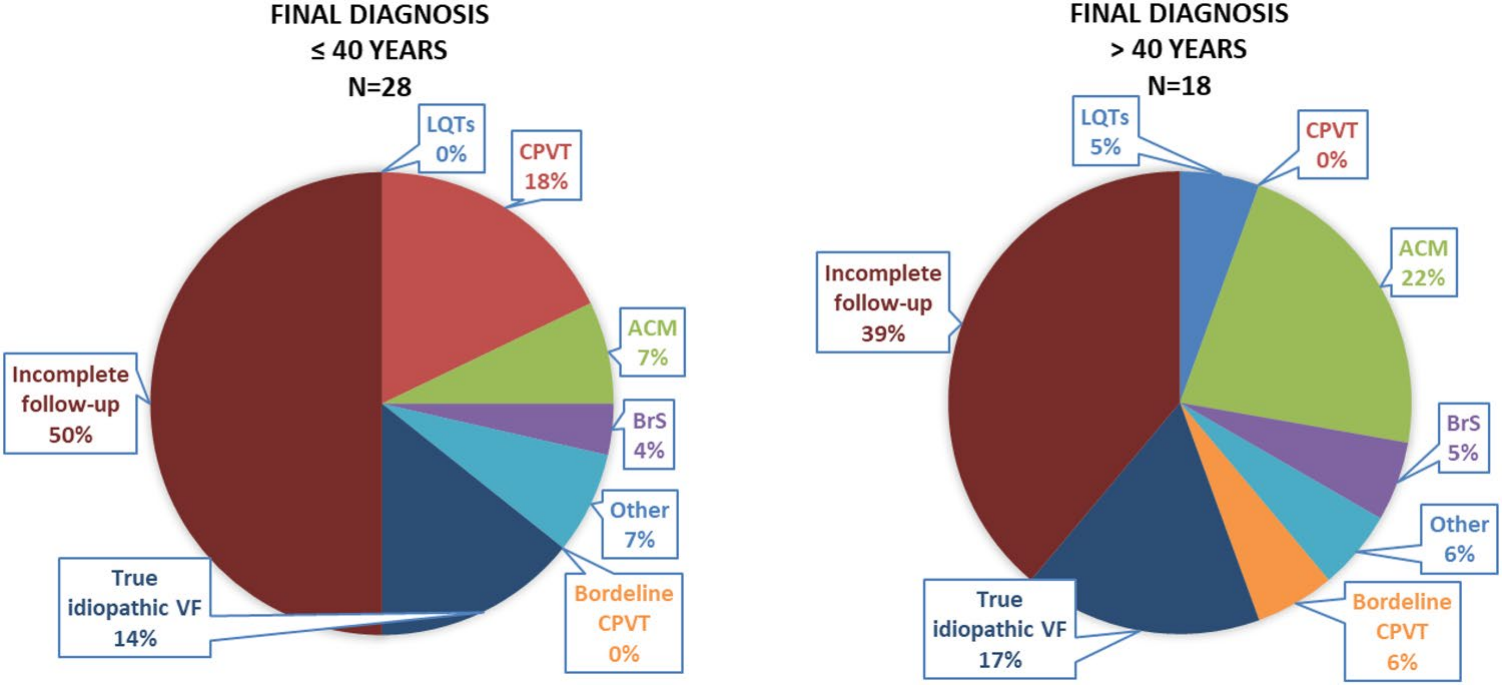
Final diagnosis according to age. No statistically significant differences were present between groups aged ≤ 40 years and aged > 40 years. In patients aged ≤ 40 years, there were trends to a higher incidence of CPVT. Incomplete follow-up group contains individuals who either refused all investigations or some step of algorithm is missing. *LQTS* long QT syndrome, *CPVT* catecholaminergic polymorphic ventricular tachycardia, *ACM* arrhythmic cardiomyopathy, *BrS* Brugada syndrome, *VF* ventricular fibrillation.

In the female patients, a statistically significantly higher incidence was observed in the category “Other” (i.e. myocarditis, amyloidosis, coronary vasospasm), as was a trend to a lower incidence of ACM (Table 2, Fig. 3). Similarly, no statistically significant differences were present between groups aged ≤ 40 years and aged > 40 years. In patients aged ≤ 40 years, there were trends to a higher incidence of CPVT and a diagnosis of true idiopathic VF (Table 2, Fig. 4).

## Discussion

ACA survivors with structurally normal hearts (i.e. normal findings on 2D-TTE and coronary angiography) were traditionally labelled as idiopathic VF cases^13^. In our survey, we are presenting the diagnostic yield of a comprehensive investigation protocol of idiopathic VF in real-life clinical settings where some investigation modalities are not always available and patient compliance is variable. Even with these modifying factors, a more specific diagnosis was established in more than one third of our patients. Mostly, the diagnostic criteria of ACM or primary electrical diseases of the heart were fulfilled. Maximizing the efforts to establish a particular diagnosis in “idiopathic” VF cases has several implications. First, a proper diagnosis can modify recommendations for the patient’s lifestyle (e.g. avoiding risk drugs in LQTS, immediate treatment of fever in BrS, and limitation of exercise activities in CPVT or ACM) and future treatment (e.g. during arrhythmic storm). Second, a diagnosis of a hereditary condition can lead to cascade family screening, which allows early diagnosis and prevention of possible life-threatening events in the relatives of ACA survivors.

The most common etiology of SCD worldwide is CAD and heart failure. These etiologies are usually absent in 5–10% of ACA survivors^5,14^. A similar proportion of patients with normal findings on 2D-TTE and coronary angiography were also identified in our study (8.9%) as these modalities were performed in 100% of our patients. At this time, it is not uncommon that, in the absence of further diagnostic work-up, an ICD is implanted^15^. This fact is critical, especially for CMR, as this investigation should be performed before ICD implantation. In the past, it was not possible to perform CMR imaging in ICD patients due to device incompatibility or because the diagnostic quality was severely limited due to device-related artifacts, even in patients with MR compatible devices. Performing CMR before ICD implantation was thus the only option, while immediate availability of this method was limited. In our group of patients, the proportion of CMR examinations reached 78.3% overall and since 2017 in our institution 100% of patients after ACA without CAD or chronic heart failure have undergone a CMR examination during initial hospitalization before ICD implantation, as it has become clear that CMR is an essential tool for ruling out possible underlying causes of ventricular malignant arrhythmias. The importance of CMR in ACA survivors is crucial as any diagnosis of ACM requires at least 1 morphological and/or structural criterion^16^. Fortunately, new mapping techniques are currently in development to increase diagnostic quality of CMR imaging to identify and quantify myocardial fibrosis in patients with implanted cardiac devices^17^. In our survey, CMR led to a specific diagnosis in 8 patients (22.2% of individuals who underwent CMR and 17.4% of the whole group)—see Fig. 1 for particular diagnoses. In the group with CMR performed, 14/36 (38.9%) patients were classified as idiopathic VF or incomplete idiopathic VF compared with 8/10 (80.0%) patients labelled as incomplete idiopathic VF in the group without CMR. The difference in results between these two groups was statistically significant (p = 0.032, Fisher’s exact test). This indicates the crucial role of CMR in the investigation algorithm. Therefore the missing CMR in 10 individuals implies that similar proportion of them might have undiagnosed cardiomyopathy. Without CMR, a potentially large group of patients with ACM, myocarditis or amyloidosis may have been misdiagnosed.

In contrast to the narrow time window for CMR, the other parts of idiopathic VF diagnostic work-up can be performed anytime in the follow-up. In this regard, it is necessary to emphasize the importance of repeated analysis of routine resting ECG. In the described group, this simple and inexpensive method led to a diagnosis of the only LQTS case and 1 of 2 BrS cases. The typical moment for ECG re-evaluation is each recurrent malignant arrhythmia in idiopathic VF patients as ECG changes can be short-term and disappear quickly.

Further recommended steps of diagnostic work-up are exercise stress testing and sodium channel blocker challenge^9^. In our survey, 13 patients refused both or one of these investigations. Again, this implies some missed cases of hereditary arrhythmic syndromes, and it emphasizes the importance of repeated offers to complete the investigation in individuals with variable compliance.

An important part of the diagnostic work-up is the exercise stress test, which is crucial for the diagnosis of CPVT and helpful in LQTS cases. In our group, this investigation led to the identification of 5 definitive CPVT and one borderline CPVT cases (13%). Recently, it has been shown that CPVT is one of the most often missed and delayed diagnoses in young ACA survivors. As Giudicessi and Ackerman^18^ observed, the reason was not only omission of this important investigation but also its misinterpretation when typical ventricular arrhythmias were induced but the CPVT diagnosis was not established. In our survey, the diagnostic yield of the exercise stress test was second to CMR (6 vs. 8 specific diagnoses identified by particular modality). The yield could have been even higher as 7 individuals of our group refused this work-up step. Interestingly, no case of LQTS was diagnosed during stress testing in our survey, although our centre has long-term experience with exercise testing in primary electrical diseases of the heart^19,20^.

For many years, sodium channel blocker challenge was a required part of the diagnostic protocol. Nevertheless, in the current version of the European guidelines it is recommended as class I only in ACA survivors^10^. Of 35 individuals indicated for this step, 25 (71.4%) consented with the challenge, which identified only 1 case of BrS. As mentioned above, the other BrS case was identified by follow-up ECG evaluation. Thus, the total proportion of BrS patients was 4.4%.

In our survey, the proportion of both structural and primary electrical diagnoses were comparable to other studies^14,21^. We observed variable compliance with the work-up protocol as 13 patients refused to undergo at least one investigation despite repeated explanations. The most common reason was fear of induced arrhythmia. This feature was not addressed in other studies.

We performed genetic testing in the true idiopathic VF patients and also in the “incomplete” idiopathic VF patients. Two P/LP variants were identified (*RYR2* gene and *FLNC* gene, respectively). Interestingly, both of these patients had normal findings on both CMR and stress testing. Genetic analysis was performed also in all patients with the “incomplete” idiopathic VF (negative both stress test and sodium channel blocker challenge but missing CMR, n = 8, see Fig. 1) without finding P/LP variants. Thus genetic analysis by itself did not lead to any particular diagnosis. Then, four VUS were identified. In the context of our current expertise, some of them are currently being analyzed in our ongoing research project of in vitro and in silico functional evaluation^22^.

## Limitations

Our survey has a certain number of limitations. The number of patients is low due to the rarity of the condition, to increase the numbers a multicentre project would be necessary. Also, this is a retrospective study of a heterogenous population recruited at a single centre. Given the retrospective nature of case selection, it is not possible to ascertain the sequence of cases. The entire diagnostic work-up was not completed in up to 30% patients: in the period from 2007 to 2017 the CMR was not available for all patients, and certain patients refused certain steps. Nonavailability of these results could have modified the possible diagnosis of true idiopathic VF. Nevertheless, in this regard, our survey reflects real-life clinical settings.

## Conclusions

Our real-life survey shows that even with an incomplete diagnostic work-up (due to unavailability of a particular method or variable patient compliance), a specific diagnosis can be identified in more than one third of the cases of idiopathic VF. Idiopathic VF is a diagnosis *per exclusionem* and, as such, it requires an active diagnostic approach. The main point of care for ACA survivors is referral to an expert centre with experience in cardiomyopathies and channelopathies. It is necessary to perform all invasive and non-invasive examinations (with emphasis on CMR prior to ICD implantation) to maximize the chance of a definitive diagnosis, with implications for treatment and family screening. In this regard, even in individuals who were missed initially or refused further investigation at the moment it is necessary to repeat the offer continuously during follow-up.

## Data Availability

The datasets generated and analyzed during the current study are not publicly available due to potentially identifiable nature but are available from the corresponding author on reasonable request.
